# Au@^109^Pd core–shell nanoparticle conjugated to trastuzumab for the therapy of HER2+ cancers: studies on the applicability of ^109^Pd/^109m^Ag in vivo generator in combined β^−^ auger electron therapy

**DOI:** 10.1186/s41181-023-00212-4

**Published:** 2023-10-11

**Authors:** Nasrin Abbasi Gharibkandi, Kamil Wawrowicz, Agnieszka Majkowska-Pilip, Kinga Żelechowska-Matysiak, Mateusz Wierzbicki, Aleksander Bilewicz

**Affiliations:** 1 Centre of Radiochemistry and Nuclear Chemistry, Institute of Nuclear Chemistry and Technology, Dorodna 16 St., 03-195 Warsaw, Poland; 2 Center for Theranostics, Jagiellonian University, Kopernika 40 St., 31-501 Cracow, Poland; 3 Institute of Biology, Warsaw University of Life Sciences, Ciszewskiego 8 St., 02-786 Warsaw, Poland; 4 Department of Nuclear Medicine, Central Clinical Hospital of the Ministry of the Interior and Administration, Wołoska 137 St., Warsaw, 02-507 Poland

**Keywords:** ^109^Pd/^109m^Ag in vivo generator, Radioimmunotherapy, Auger electron therapy, Nanotechnology

## Abstract

**Background:**

In radionuclide therapy, to enhance therapeutic efficacy, an intriguing alternative is to ensure the simultaneous implementation of low- and high-LET radiation emitted from a one radionuclide. In the present study, we introduce the concept of utilizing ^109^Pd (T_1/2_ = 13.7 h) in the form of a ^109^Pd/^109m^Ag in vivo generator. In this system, ^109^Pd emits beta particles of medium energy, while ^109m^Ag releases a cascade of conversion and Auger electrons. ^109^Pd was utilized in the form of 15 nm gold nanoparticles, which were coated with a monolayer of ^109^Pd. In this system, the ^109^Pd atoms are on the surface of the nanoparticle, while the ^109m^Ag atoms generated in the decay reaction possess the capability for unhindered emission of Auger electrons.

**Results:**

^109^Pd, obtained through neutron irradiation of natural palladium, was deposited onto 15-nm gold nanoparticles, exceeding a efficiency rate of 95%. In contrast to previously published data on in vivo generators based on chelators, where the daughter radionuclide diffuses away from the molecules, daughter radionuclide ^109m^Ag remains on the surface of gold nanoparticles after the decay of ^109^Pd. To obtain a radiobioconjugate with an affinity for HER2 receptors, polyethylene glycol chains and the monoclonal antibody trastuzumab were attached to the Au@Pd nanoparticles. The synthesized bioconjugate contained an average of 9.5 trastuzumab molecules per one nanoparticle. In vitro cell studies indicated specific binding of the Au@^109^Pd-PEG-trastuzumab radiobioconjugate to the HER2 receptor on SKOV-3 cells, resulting in 90% internalization. Confocal images illustrated the accumulation of Au@^109^Pd-PEG-trastuzumab in the perinuclear area surrounding the cell nucleus. Despite the lack of nuclear localization, which is necessary to achieve an effective cytotoxic effect of Auger electrons, a substantial cytotoxic effect, significantly greater than that of pure β^−^ and pure Auger electron emitters was observed. We hypothesize that in the studied system, the cytotoxic effect of the Auger electrons could have also occurred through the damage to the cell’s nuclear membrane by Auger electrons emitted from nanoparticles accumulated in the perinuclear area.

**Conclusion:**

The obtained results show that trastuzumab-functionalized ^109^Pd-labeled nanoparticles can be suitable for the application in combined β^−^**—**Auger electron targeted radionuclide therapy. Due to both components decay (β^−^ and conversion/Auger electrons), the ^109^Pd/^109m^Ag in vivo generator presents unique potential in this field. Despite the lack of nuclear localization, which is highly required for efficient Auger electron therapy, an adequate cytotoxic effect was attained.

**Supplementary Information:**

The online version contains supplementary material available at 10.1186/s41181-023-00212-4.

## Background

Currently used therapeutic radiopharmaceuticals implemented into the clinical practice usually consist of β^−^ or α emitters (Stokke et al. 2022). Both types of these radionuclides exhibit dissimilar properties, predisposing them to realize the treatment of different-sized lesions. Relative long tissue penetration of β^−^ particles, reaching 0.5–12 mm with LET 0.2 keV/µm, makes them favorable for large tumors treatment (Sgouros). Conversely, high-LET (50–230 keV/µm) α particles with significantly shorter tissue range (40–100 μm) are much more effective against smaller cancer sites. A wide range of β^−^ emitters applications stem primarily from their high availability. On the other hand, α emitters show great potency for individual or combined treatments, but the limited accessibility of these radionuclides hampers their application in clinical practice. Thus, there is a constant need to look for innovative therapeutic strategies that are able to deal with larger and smaller tumors simultaneously by combining different types of radiation.

In order to enhance therapeutic efficacy, an interesting alternative is to ensure simultaneous implementation of low- and high-LET radiation. The principle of this strategy is primary damage of massive tumors *via* β^−^ radiation with subsequent treatment enhancement due to the tumor subpopulations (such as stem cell-like cells), metastatic sites, or single tumors targeting with Auger electrons or α emitters (Stokke et al. 2022; Haberkorn et al. 2017). The mentioned approach is frequently used in the tandem treatment of metastatic prostate cancer, where ^177^Lu-labeled PSMA-617 is followed by ^225^Ac-PSMA-617 therapy (Khreish et al. 2020). Recently published breakthrough findings from Mueller et al. revealed that the application of ^161^Tb (T_1/2_ = 6.95 d) can induce a considerably greater therapeutic effect than observed in comparable studies with ^177^Lu (6.64 d). The uniqueness of ^161^Tb is based on the simultaneous emission of β^−^ (β_max_ ~ 550 keV) and Auger electrons (12.1 e^−^ per decay), being prevailing when compared to almost pure β^−^ emitter ^177^Lu (β_max_ = 497 keV) followed by only a negligible number of Auger/conversion electrons (1.11 e^−^ per decay) (Müller et al. 2019). This phenomenon was also confirmed by dosimetric calculations, which clearly proved the superiority of ^161^Tb over ^177^Lu in the therapeutic management of minor neoplastic lesions (< 200 μm) (Uusijärvi et al. 2006).

**Fig. 1:**
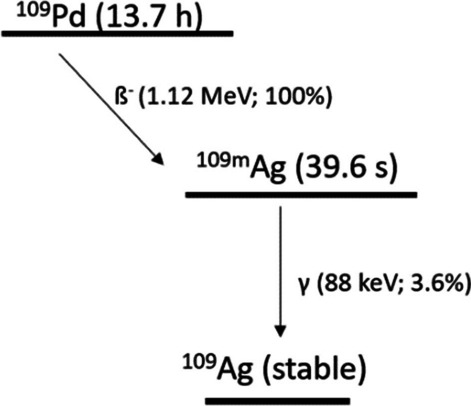
Decay scheme of ^109^Pd/^109m^Ag in vivo generator

In the present study, we introduce the idea for the application ^109^Pd (13.7 h) in the form of a ^109^Pd/^109m^Ag in vivo generator as an alternative approach to the previously described ^161^Tb. Our concept is to use β^−^ and Auger electron emitters in the form of in vivo generator, thus it follows most recent trends in radiopharmaceuticals design. This primarily includes innovative deposition pathways of different types of emitted radiation, especially as one of radionuclides is produced in vivo. Proposed in these studies ^109^Pd decays via β^−^ transition (β_max_ = 1.12 MeV, 100% yield) to ^109m^Ag (39.6 s) (Fig. 1).

Subsequently, already formed metastable intermediate decays to stable ^109^Ag which is accompanied by the emission of 88-keV photons (3.6%), followed by cascade emission of conversion and Auger electrons. Therefore, ^109m^Ag combined with ^109^Pd leads to the formation of an in vivo generator being beneficial against ^161^Tb with the greater number of Auger/conversion electrons (18 vs. 12.1) and higher β^−^ particles energy (http://www.lnhb.fr/nuclides/Pd-109_tables.pdf) (Müller et al. 2019, Müller et al. 2022). Furthermore, in contrast to ^161^Tb, ^109^Pd can be simply produced by thermal neutron irradiation. Activation of the enriched metallic Pd target (98% in ^108^Pd) for a duration of 3 days in a neutron flux of 3 × 10^13^ n/cm^2^ s results in a specific activity of 1.85 GBq/mg and nearly 100% radionuclide purity (Das et al. 2008). How easy it is to estimate this yield can be improved with a high-flux reactor, increasing specific activity up to 50 GBq/mg.

The application of radiopharmaceuticals with very high specific activity possessing is necessary for efficient Auger electron therapy. Therefore, we decided to label bioconjugates using nanotechnology. In previous studies involving ^111^In, it was found that the utilization of nanoparticles as carriers significantly enhanced the efficacy of Auger electron therapy, without compromising specificity (Sung et al. 2016; Ngo Ndjock Mbong et al. 2015; Chattopadhyay et al. 2012). In our studies, we decided to use ^109^Pd in the form of gold nanoparticles (15 nm) coated with a monolayer of ^109^Pd (Wicaksono et al. 2020). In this case, the ^109^Pd atoms will stay on the surface of the nanoparticle and the ^109m^Ag atoms which formed in the decay reaction will have the capability of unhindered emission of Auger electrons. Our particular attention was aimed to verify whether ^109m^Ag after ^109^Pd decay is able to diffuse from the NPs surface which may result in deeper intracellular penetration of its Auger electrons.

We have estimated that it is possible to deposit over 11,000 palladium atoms (assuming an atomic radius of 140 pm) onto a single gold nanoparticle surface. This could lead to a significant (300-fold) increase in specific activity compared to the method of ^111^In chelation by DOTA, as proposed by Cai et al. (Cai et al. 2016). Therefore, application of ^109^Pd-coated AuNPs (Au@^109^Pd) which we introduce in this paper should allow to obtain high specific activity of potential radiopharmaceutical with valuable radiochemical properties for combined β^−^ and Auger electron therapy. In order to enhance the precise targeting of in vivo generator, we utilized trastuzumab, a well-known monoclonal antibody specifically binding to HER2 transmembrane receptors overexpressed in tumors with HER2+ status (Tan et al. 2003).

## Methods

### Reagents

The following chemical reagents were used: gold (III) chloride trihydrate (HAuCl_4_·3H_2_O), trisodium citrate dihydrate (C_6_H_9_Na_3_O_9_), HS–PEG–COOH (poly(ethylene glycol), 5 kDa) from Sigma-Aldrich (St. Louis, MO, USA), and OPSS–PEG–NHS (alpha-pyridyl-2-disulfid-omega-carboxy succinimidyl ester poly(ethylene glycol), 5 kDa) from Creative PEGworks (Chapel Hill, NC, USA). Trastuzumab was isolated from Herceptin (Roche Pharmaceuticals, Basel, Switzerland). Iodogen (1,3,4,6-tetrachloro-3R,6R-diphenylglycouril) from Thermo Fischer Scientific (Waltham, MA, USA), and PD-10 column (GE Healthcare, Piscataway, NJ, USA). Hydrochloric acid and sodium hydroxide were purchased from POCH (Gliwice, Poland). Fluorescence mounting medium was obtained from Dako (Carpinteria, CA, USA). The following materials were also used in cell studies: McCoy’s, DMEM, MEM-EAGLE, and RPMI mediums and fetal calf serum from Biological Industries (Beth Haemek, Israel), phosphate-buffered saline (PBS), dimethylsulfoxide (DMSO), and the CellTiter 96^®^ Aqueous One Solution Reagent (MTS compound) from Promega (Mannheim, Germany). SKOV-3 and MDA-MB231 cells were also purchased from the American Type Tissue Culture Collection (ATCC, Rockville, MD, USA) and maintained based on the ATCC protocol. Additionally, all solutions were prepared using double-distilled water (18.2 MΩ·cm, Hydrolab, Straszyn, Poland).

### Radionuclides

^109^Pd was obtained by thermal neutron (1–2 × 10^14^ n/cm^2^ s) irradiation of a natural palladium target (~ 2 mg, metal powder) in the Maria nuclear reactor (Otwock-Świerk, Poland) for 1 h. After cooling for 12 h, the radioactive palladium was dissolved in 200–400 µL of aqua regia (HNO_3_:HCl—1:3) and heated at 130 °C until almost complete evaporation, with slight liquid residues to avoid sintering. Remaining nitrate [Pd(NO_3_)_2_·(H_2_O)_x_]_(x = 0 or 2)_ removal was achieved through 3 × 200 µL 0.1 M HCl portions, followed by sample heating (130 °C) until almost complete evaporation. The procedure was then repeated using dd H_2_O, finally suspending the target in the mixture of 0.1 M HCl and 0.5 M NaOH (7:1) in order to obtain palladium (II) sodium salt. The activity and radionuclidic purity of the obtained ^109^Pd were determined by gamma-ray spectrometry. Diluted solutions gained after radiochemical processing of the irradiated target were measured with an HPGe detector connected to a PC-based Multichannel Analyzer (MCA, Canberra). The 88 keV (3.67%) gamma peak was used for the determination of ^109m^Ag.

^131^I-radionuclide (no carrier added) was used for the radioiodination of trastuzumab. Na^131^I (with a specific activity of about > 550 GBq/mg) was obtained from POLATOM Radioisotope Centre Świerk, Poland. ^125^I-radionuclide (no carrier added, > 600 GBq/mg, POLATOM Radioisotope Centre Świerk, Poland) was used for the synthesis of Au@Pd^125^I-trastuzumab radiobioconjugate, which was applied for comparative cytotoxicity studies.

### Synthesis of radioactive Au@^109^Pd NPs, Au@^109^Pd -PEG-trastuzumab, and Au@Pd-^125^I-PEG-trastuzumab bioconjugates

Radioactive nanoparticles (Au@^109^Pd NPs) or their bioconjugates (Au@^109^Pd-PEG-trastuzumab and Au@Pd-^125^I-PEG-trastuzumab) were synthesized following the same procedure as described for non-radioactive compounds (Additional file 1). After completing the target material processing, ^109^Pd in the form of Na_2_^109^PdCl_4_ was used as a palladium precursor for metal deposition on the NPs surface. The previously synthesized Au@Pd-^125^I was utilized to obtain Au@Pd-^125^I-PEG-trastuzumab. The subsequent steps involving the PEGylation and conjugation with the PEG-trastuzumab bioconjugate were carried out without any modifications, as described in the Additional file 1.

### Stability studies of Au@Pd-PEG-trastuzumab bioconjugate colloid

The chemical stability of the bioconjugate in physiological conditions was measured at 37 °C for 16 days after centrifugation and dispersing the Au@Pd-PEG-trastuzumab in PBS buffer and saline (0.9% NaCl). The tendency for aggregation was investigated by measuring changes in hydrodynamic diameter and zeta potential using the Dynamic Light Scattering (DLS) technique.

Radiochemical stability assay was assessed with ɣ spectrometry in order to find ^109^Pd or ^109m^Ag detached from the core surface. For this purpose, radiobioconjugates incubated in 0.9% NaCl and human serum (HS) were centrifuged, and both separated fractions (supernatant and nanoparticles) were measured by gamma spectrometry.

### Liberation of ^109m^Ag from Au@^109^Pd NPs

Nanoparticles were incubated in water and PBS buffer (1 mM) to evaluate the liberation of ^109m^Ag, as the decay product, on Au@^109^Pd nanoparticles after β^−^ decay of ^109^Pd. The samples were centrifuged for 40 s, and the supernatant fraction was measured to find the liberated ^109m^Ag from NPs. The supernatant was measured immediately after separation by ɣ spectrometry at 88 keV, with a characteristic peak of ^109m^Ag.

### Internalization studies

Internalization studies were performed on SKOV-3 cells, as no binding was observed for MDA-MB-231 cell line (Additional file 1). Briefly, the day before the experiment, 6 × 10^5^ cells/well were seeded into 6-well plates. After completing this step, cells were washed once with PBS and tested compounds (1 mL) were added. Incubation was performed at 4 °C for 1 h in order to prevent internalization at this step. Next, medium was collected as an unbound fraction and 1 mL of fresh media was added. Plates were then incubated (37 °C, 5% CO_2_) for the desired time periods, being: 1, 6, and 24 h, respectively. To determine the membrane-bound fraction, cells were washed twice with glycine-HCl buffer (pH ~ 2.8; 0.05 M) and incubated for 5 min at 4 °C. Finally, the internalized fraction was collected after prior lysing the cells with 1 M NaOH. Non-specific binding was assessed with the same procedure as implemented for receptor binding affinity (Additional file 1).

### Confocal microscopy imaging

SKOV-3 cells (2 × 10^5^ per well) were seeded on sterile glass coverslips (diameter 12 mm, Thermo Fischer Scientific, Waltham, MA, USA) in six-well plates and incubated for 24 h. Next day, the medium was removed, and the cells were treated with trastuzumab (73 µg/mL), Au@Pd-PEG-COOH (1.62 × 10^12^ NPs/mL), and Au@Pd-PEG-trastuzumab bioconjugate (1.62 × 10^12^ NPs/mL), and incubated overnight. The applied protocol was similar to that reported previously (Dziawer et al. 2019. The cells were stained with Hoechst 33,258 (λ_ex/em_ = 352/454 nm) and an anti-human IgG secondary antibody conjugated to FITC (λ_ex/em_ = 490/525 nm), while bright-field images for nanoparticles were acquired using a transmitted light detector (T-PMT). All analysis was performed using Image J software.

### Cytotoxicity studies

Cytotoxicity experiments were conducted on SKOV-3 and MDA-MB-231 cells (3 × 10^3^ cells per well in 96-well plates) using the MTS assay. The preparation procedure was similiar as described for the binding and internalization assays. Tested non-radioactive (9-150 µg Pd/mL) and radioactive compounds in desired concentrations (180 µg Pd/mL for 40 MBq/mL; 90 µg Pd/mL for 20 MBq/mL; 45 µg Pd/mL for 10 MBq/mL) were suspended in fully supplemented growing medium and 100 µL per well was added for 24–72 h incubation. MTS reagent addition was preceded by medium exchange with one-time PBS washing as an intermediate step. The percentage of metabolically active cells was determined by addition of CellTiter96^®^ Aqueous One Solution Reagent and measurement of the absorbance at 490 nm.

### Statistical analysis

Statistical analysis, one-way ANOVA, and t-Student tests were performed with GraphPad Prism v.8 Software (GraphPad Software, San Diego, CA, USA). The results are presented as mean ± SD. p values are presented as: (*) p ≤ 0.05, (**) p ≤ 0.01, (***) p ≤ 0.001, and (****) p ≤ 0.0001.

## Results and discussion

### Production of ^109^Pd radionuclide

**Fig. 2:**
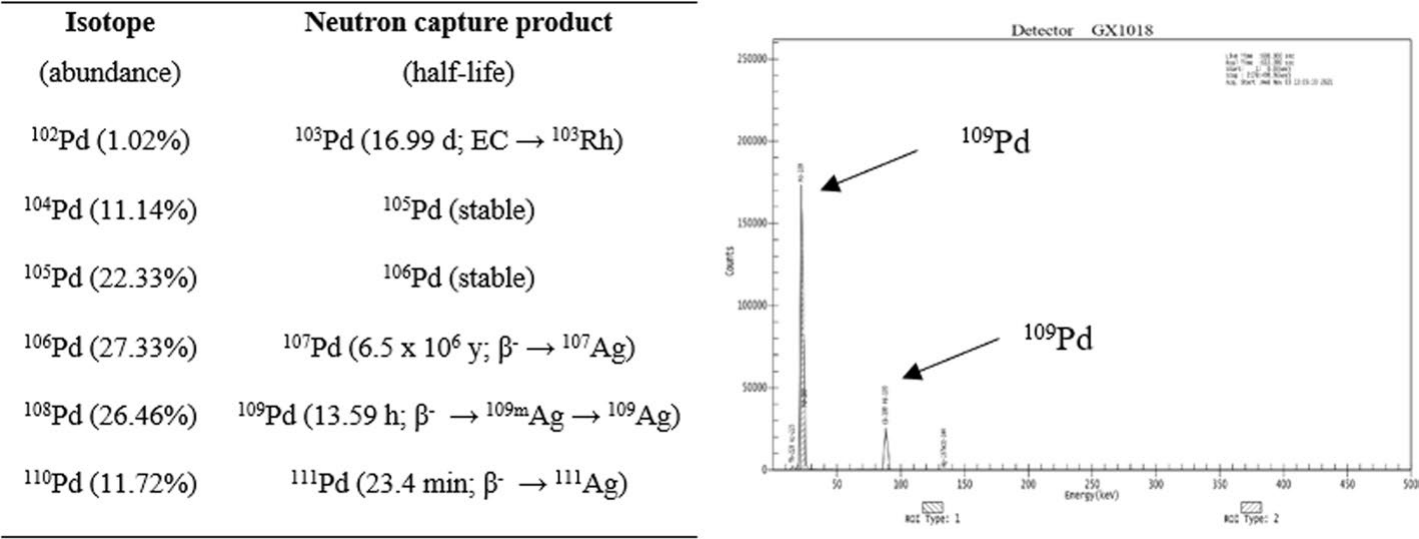
Expected radionuclides produced by thermal neutron irradiation and gamma spectrum of a 2 mg sample of natural Pd irradiated for 1 h in a neutron flux of 1–2 × 10^14^ n/cm^2^ s

One hour irradiation of 1.92 mg of natural Pd metallic target resulted in 124 MBq of total ^109^Pd activity. As we described in Fig. 2 three other Pd-radionuclides can be theoretically co-produced during the irradiation of a natural metal target. ^103^Pd production yield due to the low (~ 1%) abundance of ^102^Pd is negligible, while long-lived ^107^Pd is not produced due to a very low cross-Sect. (0.36 b). The co-produced ^111m^Pd (5.5 h; E_γ_ ~70.44 keV/7.9%) and its daughter radionuclide ^111^Ag (7.45 d; E_γ_ ~342.13 keV/6.7%) activity was insignificant due to the low cross-Sect. (0.10 ± 0.03 b) for the ^110^Pd(n,γ)^111^Pd reaction. This was confirmed *via* gamma spectrometry as no characteristic γ-rays line were found (Fig. 2). Target material processing was completed in approximately 40 min and resulted in only 5% of the initial activity loss.

### Synthesis of Au@^109^Pd NPs

**Fig. 3:**
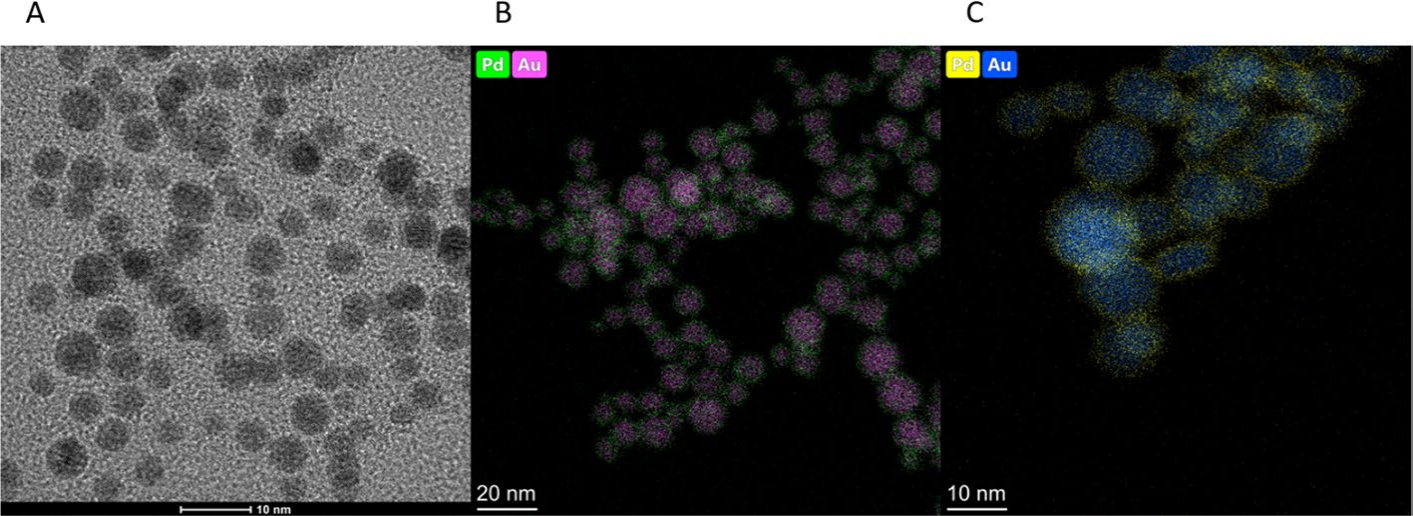
TEM image of AuNP (**A**) and HR-TEM image of Au@PdNP (**B**, **C**)

Palladium deposition on 15-nm gold nanoparticles (Fig. 3a) was performed with a high yield of over 95%. Gradual and controlled deposition of reduced palladium, followed by rapid diffusion of Pd-metal through the AuNP surface, allowed to obtain a homogenous and thin layer of radionuclide on the nanoparticle surface. High-resolution TEM images acquired in order to visualize Pd deposition confirmed its uniform distribution without any locally increased palladium clusters (Fig. 3b, c). Due to the very short tissue range of Auger and conversion electrons, covering AuNPs with a thin Pd layer was essential to ensure their effective biological activity against cancer cells. According to our calculations, it is anticipated that the resultant AuNPs will be coated with an average of 7 layers of Pd atoms. The thickness of each layer does not exceed 2 nm, and it is presumed that it should not be a barrier to the majority of the emitted Auger electrons. Additionally, with greater activities, it will be obviously feasible to diminish the thickness to a monolayer.

### Synthesis of Au@Pd-PEG-COOH and Au@Pd-PEG-trastuzumab bioconjugate

To obtain a radio-bioconjugate having affinity to HER2 receptors, the polyethylene glycol (PEG) chains and the monoclonal antibody trastuzumab have been attached to the Au@Pd nanoparticles. The synthesis was performed as reported previously for the synthesis of Au@DOTA^177^Lu-PEG-trastuzumab bioconjugate (Cai et al. 2016). PEG (ortho pyridyldisulfide-PEG-succinimidyl carboxymethyl ester; OPSS-PEG-NHS), comprising a disulfide bridge, was initially linked with the amine group of lysine in trastuzumab. After that, the conjugate was attached to the Au@Pd NPs, leading to the disruption of the disulfide bridge and the formation of robust gold-sulfur bonds. Finally, to increase the dispersity of the bioconjugate, PEGylation with HS-PEG-COOH (5 kDa) was performed.

In order to determine the mean number of attached trastuzumab molecules to a single AuNP, ^131^I-labeled trastuzumab molecules were conjugated to Au@Pd NPs (Additional file 1). Using this method, we calculated that average 9.5 trastuzumab molecules were conjugated to a single nanoparticle. This calculation was performed assuming a spherical shape for the nanoparticle with an average diameter of 15 nm, as determined by TEM, with a gold density of 19.28 g/cm³.

### Chemical and radiochemical stability studies

The chemical and radiochemical stability of synthesized radiobioconjugates was essential for considering this compound as a potential radiopharmaceutical. Nanoparticle-based compounds stability studies usually concern on the evaluation of their tendency (or not) to agglomerate in biological fluids. The hydrodynamic diameter and zeta potential of the obtained bioconjugate were measured using DLS. The results are shown in Table 1. The rise in the hydrodynamic diameter following the introduction of PEG and trastuzumab proves successful biomolecule conjugation, as in trastuzumab-modified AuNPs (Gawęda et al. 2020; Wawrowicz et al. 2021). The difference within zeta potential values of citrate-coated AuNPs and AuNP-HS-PEG-trastuzumab validates the surface modification as well. The zeta potential of AuNP-PEG-trastuzumab was − 25.1 ± 1.6 mV, providing valuable insights into the stability of AuNP-S-PEG-trastuzumab dispersion.

**Table 1:** Hydrodynamic size and zeta potential of the synthesized bioconjugate

	Au@PdNPs	Au@Pd-PEG-trastuzumab
Hydrodynamic diameter (nm)	33.65 ± 0.14	90.88 ± 0.66
Zeta potential (mV)	*− *36.9 ± 2.5	*− *25.1 ± 1.6

**Fig. 4:**
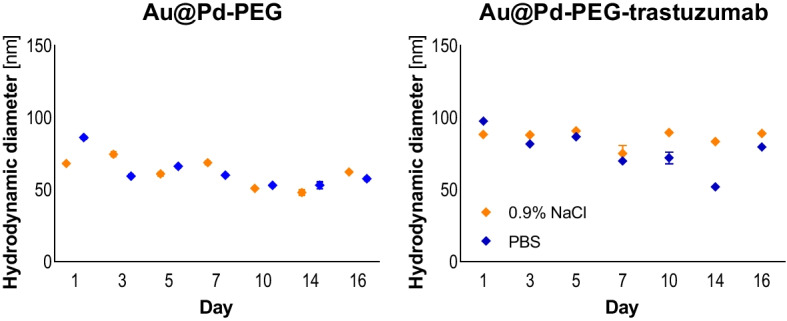
Changes in the hydrodynamic diameter of the Au@Pd-PEG (left) and Au@Pd-PEG-trastuzumab conjugates incubated in 0.9% NaCl (yellow line) and 10 mM PBS buffer (blue line) at 37 °C

As presented in Fig. 4, colloidal stability over 16 days was studied for Au@Pd-PEG and Au@Pd-PEG-trastuzumab in PBS and saline solutions. Any meaningful differences were observed between the two tested media. Our results revealed that no tendency to agglomeration or bioconjugate decomposition occurs during evaluation time, thus confirming high stability of the obtained product.

Radiochemical stability studies were performed based on γ-spectrometry, as two types of radionuclides could be potentially identified in measured samples. Any presence of ^109^Pd or its decay product (^109m^Ag) was not observed in PBS, saline, and HS solutions for at least 5 h.

### Liberation of ^109m^Ag

The possibility of a daughter radionuclide release from the carrier after nuclear decay is essential in terms of using in vivo generators in nuclear medicine. Enhanced radiation-achievable distance associated with increased tumor cell penetration may significantly improve overall therapeutic effectiveness. In our studies, no liberated ^109m^Ag was detected in water or a 1 mM PBS buffer in all analyzed samples (1 and 24 h in at least three replicates). The results show that the radionuclide ^109m^Ag formed as a result of the decay of ^109^Pd remains on the surface of AuNPs.

### Internalization*—*radiometric assay and confocal imaging

Once we confirmed, that synthesized Au@Pd-PEG-trastuzumab radiobioconjugate similarly as Au@Pt-PEG-trastuzumab (Wawrowicz et al. 2021) and Au@-PEG-trastuzumab (Żelechowska-Matysiak et al. 2023) show its ability to recognize and specifically bind to transmembrane HER2 receptors (Additional file 1), we investigated whether are they able to penetrate the cell membrane and successfully localize inside target cells. Because of the limited range of Auger electrons, radiobioconjugates internalization is essential to obtain optimal therapeutic effects. To achieve trustworthy data, we implemented two independent techniques to specify the internalization: radiometric uptake assay and confocal imaging.

Due to the limited range of Auger electrons, the internalization of radiobioconjugates is essential to attain optimal therapeutic effects. As we observed, complete (> 99%) internalization was found to occur after 1 h. This result is in agreement with our previously published papers (Gawęda et al. 2020), where similar radiobioconjugates were investigated against HER2+ breast/ovarian cancer cells. Evaluation of further time points (6 and 24 h) confirmed, that synthesized radiobioconjugates remain inside cancer cell for at least 24 h.

**Fig. 5:**
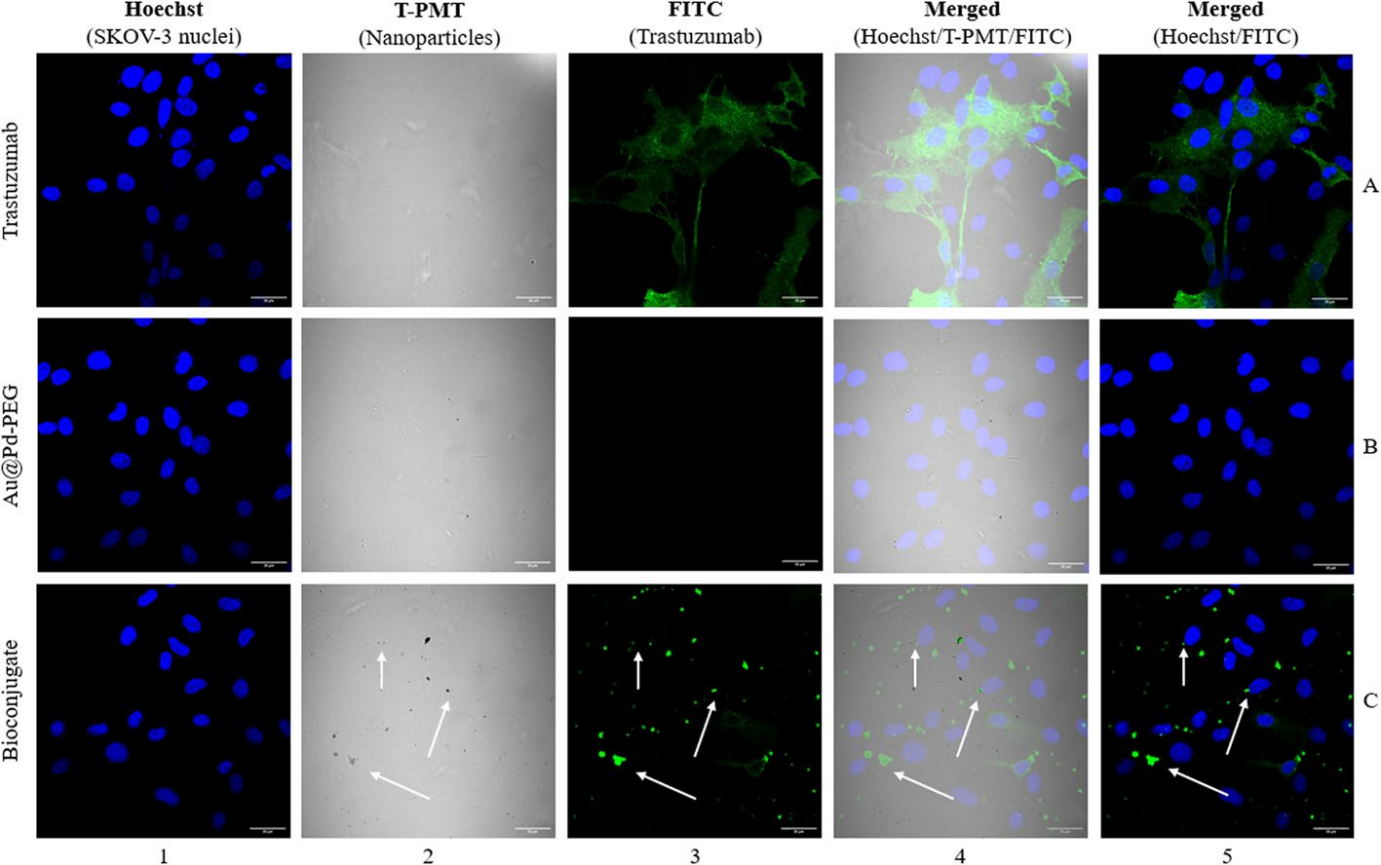
Internalization of trastuzumab, Au@Pd-PEG-COOH, and Au@Pd-trastuzumab by SKOV-3 cells determined by confocal microscopy. Fluorescence signals indicate: (green)—subcellular trastuzumab distribution; (blue)—nuclei intracellular localization. Au@Pd-containing particles (dark spots) were visualized with a transmitted light detector (T-PMT). Arrows mark the subcellular localization of the Au@Pd-trastuzumab bioconjugate

Acquired confocal images (Fig. 5) clearly indicated that only trastuzumab-conjugated NPs are able to locate inside HER2+ breast/ovarian cancer cells. As shown, PEG-ylated NPs (Au@Pd-PEG-COOH) were not detected inside the SKOV-3 cells, thus revealing the fundamental role of trastuzumab as a targeting vector. The dark spots visible on the bright background in Fig. 5, panel C/2, represent Au@Pd particles, whereas the green fluorescence (Fig. 5, panel C/3) signals indicate secondary Ab conjugated to trastuzumab being an integral part of the bioconjugate. Merged signals in Fig. 5, columns 4 and 5, demonstrated the successful penetration of the bioconjugate particles into the SKOV-3 cells and precise localization nearly to the nuclear envelope area. Hence, NPs accumulation in close proximity to the most sensitive cellular organelle allowed us to expect high cytotoxicity induced by radioactive bioconjugates.

### Cytotoxicity

**Fig. 6:**
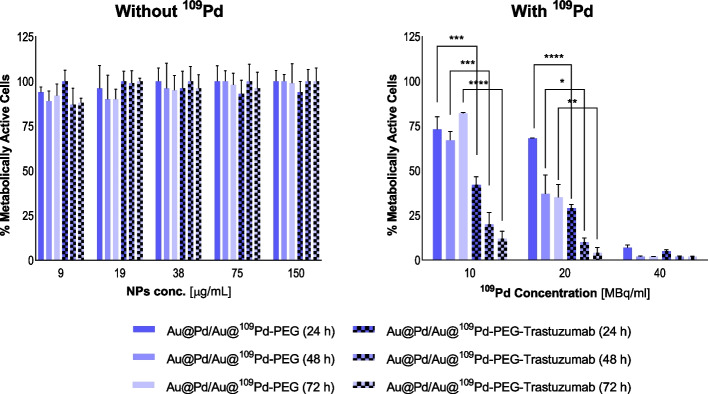
Metabolic viability of SKOV-3 cells after treatment with different concentrations of Au@Pd-PEG and Au@Pd-PEG-trastuzumab nonradioactive conjugates (left) and radioactive doses of Au@^109^Pd-PEG and Au@^109^Pd-trastuzumab conjugates (right) after 24 h, 48 h, and 72 h of treatment.

**Fig. 7:**
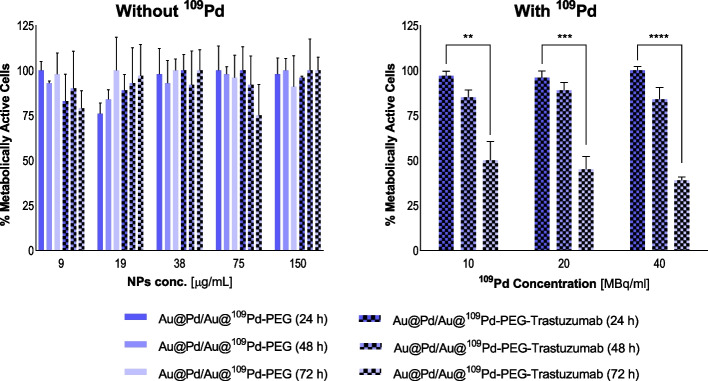
Metabolic viability of MDA-MB-231 cells after treatment with different concentrations of Au@Pd-PEG and Au@Pd-PEG-trastuzumab nonradioactive conjugates (left) and radioactive doses of Au@^109^Pd-trastuzumab conjugates (right) after 24 h, 48 h, and 72 h of treatment

Initial cytotoxicity studies concerned on non-radioactive compounds evaluation. The aim of these studies was to find out whether Au@Pd-PEG or Au@Pd-PEG-trastuzumab without ^109^Pd induce mitochondrial dysfunction leading to cell death. As shown in Figs. 6a and 7a, no significant decrease in mitochondrial activity was found with doses up to 150 µg/mL of Pd immobilized in conjugates or bioconjugates. This result is in agreement with our expectations since cold Pd nanoparticles induce a cytotoxic effect only after specific modifications (Ramalingam et al. 2020).

Metabolic viability of SKOV-3 and MDA-MB-231 (Figs. 6b and 7b) cells after exposure to diverse radioactivity doses of Au@^109^Pd-trastuzumab and Au@^109^Pd-PEG conjugates was verified at 24 h, 48 h, and 72 h.

According to the presented results, the use of Au@^109^Pd-trastuzumab radioconjugate leads to a substantial reduction in the metabolic activity of SKOV-3 cells in a dose-dependent manner. Observed changes were also found to be progressing over time. A double half-life of ^109^Pd (~ 26 h) resulted in over 50% toxicity with a slightly (*p* ≤ 0.05) stronger effect for 20 MBq/mL when compared to 10 MBq/mL. Prolonged incubation resulted in constantly decreasing the mitochondrial activity, leading after 72 h to almost complete cell death with 12.0 ± 4.3% and < 5% of unaffected mitochondrial function for 10 MBq/mL and 20 MBq/mL, respectively. Interestingly, even without the presence of trastuzumab, the Au@^109^Pd conjugate still exhibited a noticeable decrease in cell viability, observed partially for 20MBq/mL and dominantly for 40 MBq/mL. Nevertheless, the impact was much less severe than in the case of 20 MBq/mL radioboconjugates with the targeting vector. This is related to the crossfire effect of long-range β^−^ radiation, where Auger and conversion electrons exclusively affect only target cells.

Triple-negative cells (MDA-MB-231) used as reference for comparable analysis, similarly to SKOV-3 cells were usually not affected with non-radioactive conjugates. Some deviations from the overall outcome were observed, but taking into account high SD values, this cannot be considered an induced effect. For the radioactive radiobioconjugate, despite the lack of targeting and internalization, a cytotoxic effect is still apparent, although notably less pronounced compared to the SKOV-3 cell case. The observed cytotoxicity is solely attributed to the impact of long-range β^−^ radiation, where targeting and internalization are not necessary.

## Discussion

The well-established use of ^125^I and ^111^In, Auger-emitting radioisotopes, has also stimulated the search for other Auger emitters that may be more practical to use from the perspective of availability, physical half-life, and cost. In our work, we proposed a ^109^Pd/^109m^Ag in-vivo generator that combines β^−^ emission from the parent ^109^Pd radionuclide with a high emission of Auger electrons from the daughter ^109m^Ag. In our concept, the ^109^Pd/^109m^Ag in-vivo generator was applied in the form of Au@^109^Pd core–shell nanoparticles. We found that complete retention of ^109m^Ag was accomplished following the nuclear decay of the immobilized ^109^Pd on the AuNPs surface. This contrasts with previously published data for chelator-based in-vivo generators. Studies conducted on in vivo generators, such as ^103^Pd/^103m^Rh (Jensen et al. 2020), ^166^Dy/^166^Ho (Wang et al. 2022) and ^212^Pb/^212^Bi (Mirzadeh et al. 1993), have demonstrated that a significant percentage of daughter radionuclides are liberated when chelators are employed to immobilize parent radionuclides. According to (Wang et al. 2022), the loss of daughter radionuclides was attributed to their de-excitation through the internal conversion, resulting in Auger electron emission, rather than to the recoil energy associated with the emission of ß^-^ particles and neutrinos. As a result, the de-excited daughter radionuclides become highly charged what leads to electrons uptake from the surrounding chelator donor atoms. Moreover, due to the electron transfer to highly charged atoms, donor atoms of chelators acquire a positive charge. The metal-ligand bonds are then broken as a result of the repulsive force between the positively charged atoms, and the daughter radionuclides are released as free cations.

The situation changes dramatically when the parent radionuclide is immobilized on a metallic surface instead of a chelate complex. The application of a metallic surface such as AuNPs as the carrier of the mother radionuclides facilitates the availability of several delocalized electrons. After nuclear decay, highly positively charged daughter radionuclide takes electrons from neighboring Au atoms on the surface. As a result, the positive charge is rapidly transferred to the entire nanoparticle, causing only a marginal change in the surface charge. Therefore, the release of ^109m^Ag from the nanoparticles surface is not achievable. The same effect was also observed] with the ^166^Dy/^166^Ho in vivo generator deposited on the AuNPs surface (Wang et al. 2022).

This phenomenon is advantageous from the perspective of potential applications. As ^109m^Ag remains in the NPs structure, there is negligible risk of ^109^Ag unspecific accumulation in different tissues after treatment. Thus, it significantly limits the risks of post treatment side-effects related to increased and difficult to detect silver accumulation throughout the patient organism.

In performed in vitro studies, we observe the crucial role of the trastuzumab vector, which facilitates the cell internalization and cytoplasm localization of Au@^109^Pd-trastuzumab conjugate into the SKOV-3 cells. As presented in confocal images (Fig. 5), the conjugate accumulates in the perinuclear area surrounding the cell nucleus. In the case of β^−^ radiation, due to the fact that the range of β^−^ radiation emitted by ^109^Pd is a few millimeters, cell internalization is not necessary, and the β^−^ particles are capable of targeting DNA without cell internalization of ^109^Pd. This explains why we observed also cytotoxicity for Au@^109^Pd-PEG nanoparticles. Considering the low-LET characteristics of β^−^ radiation, a significantly lower DNA DSB generation yield is expected, leading to a lower overall toxicity ratio. However, the interaction of Auger electrons with the essential cell components is required to induce the cytotoxic effect correlated with the Auger electron emission from ^109m^Ag as the daughter radionuclide. Cellular DNA is typically considered to be the most sensitive target of Auger electron emitters. The double-stranded DNA helix possesses a diameter of 2 nm. During a typical Auger emission, the greatest release of energy happens in 1–2 nm spheres around the decay site (Buchegger et al. 2006). Similar to the α-radiation path through the cell nucleus, the loss of genetic information occurs in these double-strand breaks, which is attributed to the nucleotide breakdown on both strands (Lobachevsky, et al. 2000). For ^125^I decays associated with DNA, it means “One decay = one double-strand break” (Elmroth et al. 2005). Furthermore, as has been proposed in some publications, since the cell membrane plays a key role in cell viability, the effects of Auger electrons generated by membrane-bound radiolabeled mAbs should not be overlooked (Pouget et al. 2008; Paillas et al. 2016, Muller et al. 2022). Considering the significant accumulation of nanoparticles on the nuclear membrane within the range of Auger and conversion electron interaction as depicted in confocal microscopy images (Fig. 5), it has been concluded that nuclear envelope damage caused by Auger electrons may be one of numerous reasons of cell death. In order to verify the therapy enhancement for simultaneous β^−^ particle emission and conversion/Auger electrons, we decided to perform a comparable analysis with ^125^I (Auger) and ^198^Au (β^−^) radionuclides. Both of them were directly implemented into the nanoparticle structure by chemisorption of iodine (^125^I-labeled Au@Pd-trastuzumab (Additional file 1) or by using radioactive ^198^AuNPs-trastuzumab radiobioconjugates, as we previously described (Żelechowska-Matysiak et al. 2023).

**Table 2:** Comparison of cytotoxicity of radiobioconjugates labeled with Auger electron emitter (^125^I), β-emitter (^198^Au) and Auger and β-electron emitter (^109^Pd/^109m^Ag in vivo generator)

Radiobioconjugate	T_1/2_ (days)	Decay mode	Decay numer in 48 h	Radiation emitted	% of metabolic activity in MTS test
Au@Pd^125^I-trastuzumab	59.49	EC	3.42 × 10^12^	Auger, low energy gamma	60
^198^Au-trastuzumab	2.69	β^−^	2.70 × 10^12^	β^−^_avg_ (315 keV)	53
Au@^109^Pd-trastuzumab	0.57	β^−^, IT	1.30 × 10^12^	β^−^_avg_ (436 keV) + Auger/conversion	10

Activity concentration: 20 MBq/ml, incubation time: 48 h

The comparison provided in Table 2 is undoubtedly an approximation. It does not take into account several parameters, including radionuclide half-life, radiation intensity and energy. In addition, the emitted γ radiation was not taken into account, considering its insignificance in relation to the corpuscular radiation.We intended to compare the obtained data with currently existing alternative pathways. The acquired results, however, clearly reveal that the cytotoxicity of the ^109^Pd/^109m^Ag in vivo generator-based conjugate (β^-^ and Auger electron emitter) is significantly higher than that of the conjugates radiolabeled with the same activities of β^−^ or Auger electron emitters. This effect will be much greater if the considerably shorter half-life of ^109^Pd is taken into account.

## Conclusion

The ^109^Pd/^109m^Ag in vivo generator in the form of trastuzumab conjugated core–shell Au@^109^Pd nanoparticles used in this paper generated significantly better in vitro cytotoxicity results than Au@PdNPs labeled with either ^125^I (Auger emitter) or ^198^Au (β^−^ emitter). Due to both components decay (β^−^ and conversion/Auger electrons), the ^109^Pd/^109m^Ag in vivo generator presents unique potential in this field. Despite the lack of nuclear localization, which is highly required for efficient Auger electron therapy (Costantini et al. 2010), an adequate cytotoxic effect was attained.

We assume that the toxic effect could have been induced apart of β^−^ particles by Auger/conversion electrons emitted from the nanoparticles in the perinuclear area efficiently damaging the nuclear envelope. Therefore, radiobioconjugates labeled with the ^109^Pd/^109m^Ag radionuclide generator might show the advantages of both β^−^ emitters (a few millimeters range of radiation, crossfire effect) and Auger electrons (large LET, double-stranded DNA breaks). Therefore, it can be expected, that ^109^Pd/^109m^Ag-labeled vectors may have a more substantial antitumor performance, when used either for the treatment of small tumor clusters or targeting of medium-sized tumors.

Nonetheless, the development of this concept requires clarifying its disadvantages, limitations and challenges that need to be overcome. Due to the quite large size and considerable accumulation of the radiolabeled nanoconstructs in the liver, lungs, and spleen, as well as fast blood clearance, toxic radiation exposure of these organs, and reduced tumor uptake, intravenous administration route of Au@^109^Pd-trastuzumab is excluded. One possible concept which can be implemented is strictly localized administration i.e., directly into the tumor or post-surgery resection cavity. This strategy can also be investigated for the synthesis of Au@^109^Pd nanoparticles conjugated with other different biological vectors, which, in turn, would broaden the applicability of this approach for targeting a diverse variety of tumors. Nevertheless, the presented concept and obtained results are highly promising and encouraging for further development of the described treatment strategy.

### Supplementary Information

**Additional file 1.** Supplementary information.

## Data Availability

Data is available upon reasonable request to the corresponding author.
