# Supplementary material for: Au@109Pd core–shell nanoparticle conjugated to trastuzumab for the therapy of HER2+ cancers: studies on the applicability of 109Pd/109mAg in vivo generator in combined β− auger electron therapy
Source: EJNMMI Radiopharm Chem. 2023 Oct 11;8:26. doi: 10.1186/s41181-023-00212-4 (PMC10567614; doi:10.1186/s41181-023-00212-4)
Supplement: Supplementary file 1 — Additional file 1. Supplementary information. [file 41181_2023_212_MOESM1_ESM.docx]

**Au@^109^Pd core-shell nanoparticle conjugated to trastuzumab for the therapy of HER2+ cancers. Studies on the applicability of ^109^Pd/^109m^Ag *in vivo* generator in combined β^-^ Auger electron therapy**

**Supplementary information**

**Materials and methods**

*Synthesis and Characterization of Au@Pd (15 nm)*

The synthesis of gold nanoparticles was conducted in an aqueous solution based on the procedures described by Turkevich et al [^[[1]](#endnote-1)^], slightly modified by our group. Core-shell nanoparticles synthesis was performed regarding to published by Wawrowicz et al [^[[2]](#endnote-2)^] with using Na_2_PdCl_4_ (0.27 mg, 1 mM) or Na_2_^109^PdCl_4_ as palladium precursor and ascorbic acid (7.5 mg, 2M) as reducing agent. Briefly, 55 mL of 15 AuNPs was heated to 90^o^C (260 rpm) for 15 minutes. After that, first portion of Na_2_^109^PdCl_4_ and ascorbic acid were added into the solution mixture. Subsequent three parts of Na_2_^109^PdCl_4_ were added with 10 min intervals, while reducing agent (8 portions in total) was added in 30 min intervals. Each portion of relevant reagent (either Pd precursor or AA) was divided into equal volume parts in order to ensure homogenic conditions of synthetic process. During whole procedure, 90^o^C and 260 rpm were applied. After addition of last ascorbic acid portion, mixture was heated and mixed for another 30 minutes, cooled down and stored in 4^o^C in glass flask covered with aluminium foil.

*Synthesis of Au@Pd-^125^I (15 nm)*

*Au@Pd* nanoparticles were centrifuged for 10 min at 10 000 rpm and redispersed in deionized water. Subsequently, ^125^I was added and reaction was kept in room temperature for 1 hour with continuous mixing. Finally, HS-PEG-COOH (MW = 5 kDa) was added with desired excess and reaction was continued for the next 30 min.

*Nanoparticles Characterization*

HR-TEM Microscopy (TALOS™ F200X, Thermo Fisher Scientific - Waltham, MA, USA) was used to determine the size, shape, and collocation of the palladium layer on nanoparticles. The hydrodynamic size and zeta potential of the nanoparticles were obtained by dynamic light scattering analysis (DLS, Malvern, UK). The radioactivity level of ^109^Pd and ^131^I samples was measured using the Wizard^2^ Gamma Counter (PerkinElmer, Waltham, MA, USA). The Thin Layer Chromatography technique with the Storage Phosphor System Cyclone Plus (PerkinElmer, Waltham, MA, USA), glass microfiber chromatography paper impregnated with silica gel (iTLC SG, Agilent Technologies, Santa Clara, CA, USA), and PBS buffer as the mobile phase were used to monitor the stability of the ^131^I-radiolabeled trastuzumab during synthesis.

*Synthesis of Au@Pd-PEG-COOH and* *Au@Pd-PEG-trastuzumab Bioconjugate*

PEGylated core-shell gold nanoparticles were generated by the addition of HS-PEG-COOH (15000 molar excess) and stirring for 30 minutes at room temperature. Bioconjugation was conducted as described by Cai et al. [^[[3]](#endnote-3)^]. Trastuzumab (1 mg) was mixed with a 25-molar excess of OPSS-PEG-NHS (5 kDa) in carbonate buffer (100 mM). The reaction was carried out overnight, and the purification of OPSS-PEG-Trastuzumab was then conducted using centrifugal concentrators Vivaspin®500 100 kDa cut-off (Sartorius, Goettingen, Germany) to remove the unreacted OPSS-PEG-NHS. After that, in Protein LoBind Tubes (Eppendorf, Hamburg, Germany), 48 μg of OPSS-PEG-trastuzumab was reacted with 2 mL of previously purified and concentrated Au@Pd in 20 mM carbonate buffer (pH~8.90; 0.02M) for 45 minutes. In the final step, a 15000-molar excess of HS-PEG-COOH (5 kDa) was added to increase the dispersity of bioconjugates (30 min, RT). The obtained product was finally purified by centrifuging (11000 rpm), dispersed in deionized water, and characterized.

*Determination of Conjugated Trastuzumab Molecules to Au@Pd Nanoparticles*

To estimate the number of conjugated molecules of Trastuzumab to nanoparticles, iodinated Trastuzumab ([^131^I]trastuzumab) was used as described before [2]. Briefly, 2.2 mg of trastuzumab, 60 MBq of ^131^I, and PBS buffer (0.1M) were mixed in an Iodogen precoated iodination tube for 10 minutes at room temperature. The reaction mixture was further purified using a Sephadex G-25 PD-10 column and 10 mM PBS buffer as a mobile phase. Buffer exchange was performed using 100,000 MWCO Vivaspin®500 centrifugal concentrators, and the final product was dissolved in water. After that, 2 mg of [^131^I]trastuzumab was reacted overnight with a 25-fold molar excess of OPSS-PEG-NHS (5 kDa) as described above for non-radioactive synthesis. To estimate the conjugation yield, the activity of both the pellet containing nanoparticles and the supernatant fractions was measured after centrifugation of nanoparticles. The number of attached iodinated trastuzumab molecules to Au@Pd was finally determined by dividing the moles of protein by the moles of nanoparticles.

*Receptor Binding Studies*

To evaluate the receptor binding affinity of the Au@109Pd-PEG-Trastuzumab bioconjugate, SKOV-3 and MDA-MB-231 cells were cultured in McCoy's 5A and DMEM Modified mediums supplemented with 10% fetal bovine serum and 1% penicillin-streptomycin. The cells were seeded into six-well plates (6 x 10^5^/well) and incubated (37°C, 5% CO_2_) overnight. The next day, the cells were rinsed with PBS once, and then different concentrations of bioconjugate (in one mL) were added to the wells for 1.5 h incubation. After completing the incubation step, medium was collected and the cells were washed once with PBS as unbound fraction. Subsequently, using 1M NaOH cells were lysed and collected in order to calculate the percentage of bound fraction. All of the fractions were measured using the Wizard^2^ Detector Gamma Counter. For evaluation of non-specific binding, 100-molar excess of antibody was used to saturate HER2 receptors in parallel experiment.

**Results**

*Determination of Conjugated Trastuzumab Molecules to Au@Pd Nanoparticles*

In order to determine the mean number of attached trastuzumab molecules to a single Au@PdNP, ^131^I-labeled trastuzumab was integrated with Au@Pd NPs. Through this method, the number of 9.5 trastuzumab molecules was conjugated with a single nanoparticle. This calculation was performed assuming a spherical shape for the nanoparticle with an average diameter of 15 nm, as determined by TEM, with gold density of 19.28 g/cm³.The hydrodynamic diameter and zeta potential of the obtained bioconjugate were measured using DLS. The rise in the hydrodynamic diameter following the introduction of PEG and trastuzumab proves successful biomolecules conjugation, as in trastuzumab-modified AuNPs [^[[4]](#endnote-4)^,^[[5]](#endnote-5)^]. The difference within zeta potential values of citrate-coated Au@PdNPs and Au@Pd-PEG-trastuzumab validates the surface modification as well. The zeta potential of AuNP-PEG-trastuzumab was -25.1 ± 1.6 mV, providing valuable insights into the stability of AuNP-S-PEG-trastuzumab dispersion. The results indicate there is a repulsive force between the particles and there is no tendency of aggregation.

*Receptor affinity studies*

- - 1. *Receptor affinity studies*

The affinity of the radiobioconjugate to HER2 receptors was examined on SKOV-3 (HER2+) cell line with the use of radioactive Au@^109^Pd-trastuzumab applying the same procedure as for other nanoparticle-trastuzumab radiobioconjugates [4,5]. We observed significant differences of binding in the presence and in the absence of free trastuzumab used in 100-fold molar excess to block the receptors. The results presented in Fig. 1. clearly indicate that the Au@^109^Pd-trastuzumab bioconjugate binds specifically to the HER2 receptor on SKOV-3 cells. The specific binding values obtained for Au@^109^Pd-trastuzumab are very similar to those of the Au@Pt-[^131^I]trastuzumab conjugates we have previously studied (both determined under the same conditions), although the Au@Pt-[^131^I]trastuzumab radiobioconjugate was based on nanoparticles twice as large in diameter (30 nm) [2]. Also, a comparison of the specific binding affinity for Au@^109^Pd-trastuzumab bioconjugate with the specific binding affinity for [^131^I]trastuzumab molecules [17] indicates that attachment of 15 nm AuNPs reduces the receptor-specific affinity of a trastuzumab molecule to a small degree. For triple-negative breast cancer cells (MDA-MB-231), that do not overexpress Her2 receptors, no specific binding of the conjugate was observed.

Fig. 1. Specific binding of Au@^109^Pd-trastuzumab on SKOV-3 cell line.

*Cytotoxicity of Au@Pd-^125^I-PEG-trastuzumab*

To compare the cytotoxicity of the Au@^109^Pd-trastuzumab bioconjugate, which emits both β^−^ particles and Auger electrons simultaneously, with that of Au@Pd-^125^I-trastuzumab bioconjugate (Auger electrons emitter), SKOV-3 cell survival tests were conducted using the MTS assay. The results are shown in Fig. 2.

Fig.2. Metabolic viability of SKOV-3 cells after treatment with different concentration Au@Pd-^125^I-PEG-trastuzumab.

As shown in Fig.2, similarly to the ^109^Pd-labeled conjugate, the use of the Au@Pd-^125^I-trastuzumab radioconjugate leads to a substantial reduction in the metabolic activity of SKOV-3 cells in a dose-dependent manner. The observed changes also decreased over time. However, the % of metabolically active cells is significantly higher after treatment with Au@Pd-^125^I- PEG-trastuzumab than with Au@^109^Pd-PEG-trastuzumab. This clearly indicates a stronger cytotoxic effect of the conjugate labeled with a ^109^Pd emitting β^−^ and Auger radiation.

1. . Turkevich J, Stevenson PC, Hillier J. A study of the nucleation and growth processes in the synthesis of colloidal gold, Discuss Faraday Soc. 1951;11:55–75. 5 [↑](#endnote-ref-1)
2. . Wawrowicz K, Majkowska-Pilip A, Gaweł D, Chajduk E, Pieńkowski T, Bilewicz A. Au@Pt Core-Shell Nanoparticle Bioconjugates for the Therapy of HER2+ Breast cancer and hepatocellular carcinoma. model studies on the applicability of ^193m^Pt and ^195m^Pt radionuclides in Auger electron therapy. Molecules 2021;26: 2051-71. [↑](#endnote-ref-2)
3. . Cai Z, Chattopadhyay N, Yang K, Kwon YL, Yook S, Pignol J-P, Reilly RM. ^111^In-labeled trastuzumab-modified gold nanoparticles are cytotoxic in vitro to HER2-positive breast cancer cells and arrest tumor growth in vivo in athymic mice after intratumoral injection. Nucl Med Biol. 2016;43:818–26. [↑](#endnote-ref-3)
4. . Gawęda W, Pruszyński M, Cędrowska E, Rodak M, Majkowska-Pilip A, Gaweł D, Bruchertseifer F, Morgenstern A, Bilewicz A. Trastuzumab modified barium ferrite magnetic nanoparticles labeled with radium-223: A new potential radiobioconjugate for alpha radioimmunotherapy. Nanomaterials 2020;10:2067-88. [↑](#endnote-ref-4)
5. . Dziawer Ł, Majkowska-Pilip A, Gaweł D, Godlewska M, Pruszyński M, Jastrzębski J, Wąs B, Bilewicz A, Trastuzumab-modified gold nanoparticles labeled with ^211^At as a prospective tool for local treatment of HER2-positive breast cancer. Nanomaterials 2019;9:632-47. [↑](#endnote-ref-5)
